# Real‐World Effectiveness and Safety of a Tubeless Automated Insulin Delivery System With an Adaptive Control Algorithm (APGO) in Children and Adolescents With Type 1 Diabetes: A Multicentre Observational Study

**DOI:** 10.1111/dom.71101

**Published:** 2026-07-23

**Authors:** Angela Zanfardino, Dario Iafusco, Giorgia Ippolito, Emanuele Miraglia del Giudice, Assunta Serena Rollato, Antonietta Chianese, Adele Figuccia, Sonia Toni, Annalisa Giulia Ferlito, Barbara Piccini

**Affiliations:** ^1^ Regional Centre for Pediatric Diabetes, Department of Pediatrics University of the Study of Campania Naples Italy; ^2^ Department of Precision Medicine in Medical, Surgical and Critical Care (Me.Pre.C.C.) University of Palermo Palermo Italy; ^3^ Diabetology Unit Meyer Children's Hospital IRCCS Florence Italy

**Keywords:** A8 TouchCare Nano system, artificial pancreas, automated insulin delivery (AID), diabetes technology, hybrid closed‐loop, insulin pump

## Abstract

**Aim:**

Automated insulin delivery (AID) systems improve glycaemic outcomes in Type 1 diabetes; however, paediatric real‐world data on newly introduced tubeless AID systems remain limited. We aimed to provide the first clinical evaluation of the A8 TouchCare Nano AID system with the APGO algorithm in children and adolescents with Type 1 diabetes.

**Materials and Methods:**

In this prospective observational study, children and adolescents initiating TouchCare Nano AID therapy were followed for 6 months. Continuous glucose monitoring metrics were assessed at baseline and 1, 3 and 6 months. The primary outcome was change in time in range (TIR, 70–180 mg/dL). Secondary outcomes included time in tight range (TiTR, 70–140 mg/dL), time above and below range, glucose variability, insulin dose, achievement of ISPAD glycaemic targets and safety. HbA1c was measured at baseline, 3 and 6 months.

**Results:**

Seventy‐six participants (mean age 13.3 ± 3.2 years) completed follow‐up. Mean TIR increased from 59.0% ± 15.6% at baseline to 74.2% ± 8.9% at 1 month and remained significantly higher at 3 and 6 months (all *p* < 0.05). TiTR significantly improved, whilst time above range decreased and time below range remained stable. More than half of participants achieved the ISPAD target of TIR > 70% at 6 months. Mean sensor glucose and glucose variability declined, and HbA1c decreased from 7.4% ± 0.8% to 6.9% ± 0.7% at 3 and 6 months (all *p* < 0.05). No episodes of severe hypoglycaemia or diabetic ketoacidosis were observed.

**Conclusions:**

In routine paediatric care, use of this tubeless AID system was associated with rapid and sustained improvements in glycaemic control without increased hypoglycaemia.

**Trial Registration:** Clinicaltrials.gov: NCT07425912.

## 1 Introduction

Automated insulin delivery (AID) systems have revolutionised the management of type 1 diabetes by integrating continuous glucose monitoring (CGM), insulin pump technology, and advanced control algorithms to optimise glycaemic outcomes, reduce the daily burden of diabetes management and potentially lower the risk of long‐term microvascular and macrovascular complications [1, 2].

As observed in patient‐reported outcome studies [3], patch pumps are often appreciated by patients for their tubeless and wearable design. These devices have undergone substantial technological advances and have progressively become fully integrated AID systems. Several studies have demonstrated significant improvements in glycaemic outcomes with tubeless AID systems in both clinical trial and real‐world settings [4, 5]. These benefits have also been consistently observed in paediatric cohorts, in which early real‐world adoption has been associated with rapid improvements in glucose stability and a low incidence of hypoglycaemia following the transition from previous therapies [6, 7].

The A8 TouchCare Nano system (Medtrum Technologies Inc., Shanghai, China) is a tiny patch pump designed to operate in a fully automated advanced closed‐loop mode when integrated with its corresponding CGM [8, 9]. The device incorporates a new adaptive, artificial intelligence–based control algorithm (APGO) that learns from individual glycaemic patterns over time to optimise insulin delivery.

Whilst the reliability and clinical performance of previous generations of the TouchCare patch pump system have been documented in an earlier clinical evaluation [10], and preliminary real‐world data on the TouchCare Nano system incorporating the APGO algorithm have recently been reported in adults [11, 12], to the best of our knowledge, no studies have yet evaluated the efficacy and safety of this system in a real‐world paediatric setting.

This study aims to provide real‐world clinical evidence on the efficacy and safety of the TouchCare Nano system with the APGO algorithm in a paediatric cohort with T1D.

The primary objective of this study was to evaluate time in range (TIR, 70–180 mg/dL; 3.9–10.0 mmol/L) at 1, 3 and 6 months following the transition to AID therapy, compared with baseline values obtained during previous treatment modalities, including multiple daily injections (MDI) or sensor‐augmented pump (SAP) therapy. Secondary objectives, assessed at the same time points, included CGM‐derived glucose metrics, pump‐related parameters, ISPAD glycaemic targets [13] and clinical variables (glycated haemoglobin and BMI). Safety outcomes, specifically the incidence of diabetic ketoacidosis (DKA) and severe hypoglycaemia, were systematically monitored throughout the 6‐month follow‐up period.

## 2 Materials and Methods

### 2.1 Data Source and Subjects

This prospective multicentre observational study included a cohort of consecutively enrolled patients with T1D recruited from two specialised Italian centres: the Regional Reference Centre for Paediatric Diabetology at the AOU University of Campania ‘Luigi Vanvitelli’ in Naples, and the Department of Diabetology at the AOU Meyer IRCCS in Florence. Baseline was defined as the initiation of AID therapy, which was funded by the Italian National Health Service (Servizio Sanitario Nazionale, SSN). Participants were followed for 6 months (from September 2025 to March 2026). After completion of the study, all participants continued using the system within routine clinical care. The study protocol was approved by the Ethics Committee of the University of Campania ‘Luigi Vanvitelli’ and was conducted in accordance with the principles of the Declaration of Helsinki. Written informed consent was obtained from all participants and their parents or legal guardians before study participation. The decision to initiate AID therapy was based exclusively on routine clinical practise and was independent of the study protocol, which did not influence therapeutic management.

Eligibility criteria included age between 6 and 18 years, T1D duration of at least 6 months, and continuous use of insulin therapy—either MDI or SAP—for at least 6 months before enrolment. Patients were excluded if they had experienced episodes of DKA within the 3 months preceding enrolment, were receiving medications known to interfere with glucose metabolism, weighed less than 20 kg or required a total daily insulin dose of less than 10 IU, as these conditions precluded safe transition to Auto Mode.

Throughout the study, all participants used the A8 TouchCare Nano system (Medtrum Technologies Inc., Shanghai, China), which consists of the A8 glucose sensor, a patch pump (Nano) and the APGO algorithm. APGO is a hybrid closed‐loop system based on Model Predictive Control–Proportional Integral Derivative (MPC–PID) technology that automatically adjusts basal insulin delivery and administers correction boluses. Automated insulin delivery is influenced by user‐defined settings, including insulin sensitivity factor and glucose targets, and the system can deliver up to 30 correction boluses per hour. Automated mode is interrupted if sensor glucose data are unavailable for > 3 h, if maximum insulin delivery (approximately twice the daily basal dose) occurs over 8 h during persistent hyperglycaemia, or if only minimal insulin delivery (approximately one‐quarter of the daily basal dose) occurs over 5 h. In these situations, the system automatically switches to PLGS (Predictive Low Glucose Suspend) mode.

Following system initiation at baseline (Day 0), participants underwent a brief run‐in period in PLGS mode lasting no more than 7 days, after which they transitioned to Auto Mode. Auto Mode settings included a target glucose level of 110 mg/dL (6.1 mmol/L), an active insulin time of 2 h and activated corrective boluses.

CGM and insulin pump data were analysed using EasyView Pro, LibreView or Dexcom Clarity at baseline, and EasyView Pro at follow‐up, using data from the last 15 days of observation. All patients and caregivers received standard education regarding CGM interpretation and were instructed to perform capillary glucose verification whenever sensor values appeared inconsistent with symptoms or clinical expectations. They were also instructed to perform at least two capillary glucose measurements daily at different times. These records were reviewed by the diabetes care team during follow‐up visits.

### 2.2 Outcome Variables

Clinical variables, including HbA1c and BMI z score, were collected at baseline and at 3 and 6 months after transition to AID therapy. CGM‐derived metrics and insulin delivery parameters were assessed at all follow‐up visits (1, 3 and 6 months) and included: TIR (70–180 mg/dL; 3.9–10.0 mmol/L), time in tight range (TiTR 70–140 mg/dL; 3.9–7.8 mmol/L), time above range (TAR > 180 mg/dL; > 10.0 mmol/L ~ TAR1 181–250 mg/dL; 10.1–13.9 mmol/L ~ TAR2 > 250 mg/dL; > 13.9 mmol/L), time below range (TBR < 70 mg/dL; < 3.9 mmol/L ~ TBR1, 54–69 mg/dL; 3.0–3.8 mmol/L ~ TBR2 < 54 mg/dL; < 3.0 mmol/L), glucose management indicator (GMI), coefficient of variation (CV), mean sensor glucose and glucose standard deviation (SD). Furthermore, the total daily insulin dose—categorised into basal and bolus delivery, including corrective doses—and the percentage of time spent in Auto‐Mode were systematically recorded at each study visit.

### 2.3 Statistical Analysis

Continuous variables are reported as mean ± SD or median (IQR), as appropriate and categorical variables as frequencies and percentages. Normality was assessed using the Shapiro–Wilk test. Changes across baseline, 1‐, 3‐ and 6‐month follow‐up visits were evaluated using repeated‐measures ANOVA for normally distributed variables or the Friedman test for non‐normally distributed variables. When global tests were significant, post hoc pairwise comparisons were performed using appropriate paired tests with Bonferroni or Holm correction for multiple comparisons. Paired‐samples proportion tests were used to compare percentages over time. A two‐sided *p*‐value < 0.05 was considered statistically significant. To assess the relationship between improvements in TiTR and overall glycaemic control, Pearson correlations were calculated between changes (Δ; follow‐up minus baseline) in TiTR and TIR at 1, 3 and 6 months.

To evaluate treatment effects across clinically relevant subgroups, changes from baseline in HbA1c, TIR and TiTR were compared according to baseline HbA1c (< 8% vs. ≥ 8%), age (< 12 vs. ≥ 12 years) and previous treatment modality (MDI vs. SAP). Between‐group comparisons were performed using the independent‐samples *t*‐test or Mann–Whitney *U* test, as appropriate, with Holm correction for multiple testing.

An a priori sample size calculation for the primary outcome (TIR) was performed using repeated‐measures ANOVA, assuming an effect size of 0.25, four measurements, *α* = 0.05 and 90% power, yielding a minimum required sample size of 49 participants. Post hoc power analysis based on observed data (*f* = 0.92; partial *η* ^2^ = 0.46) showed > 99% power with the final sample of 76 participants.

All analyses were performed using R software (version 4.3.1) and G*Power.

## 3 Results

### 3.1 Population

**FIGURE 1:**
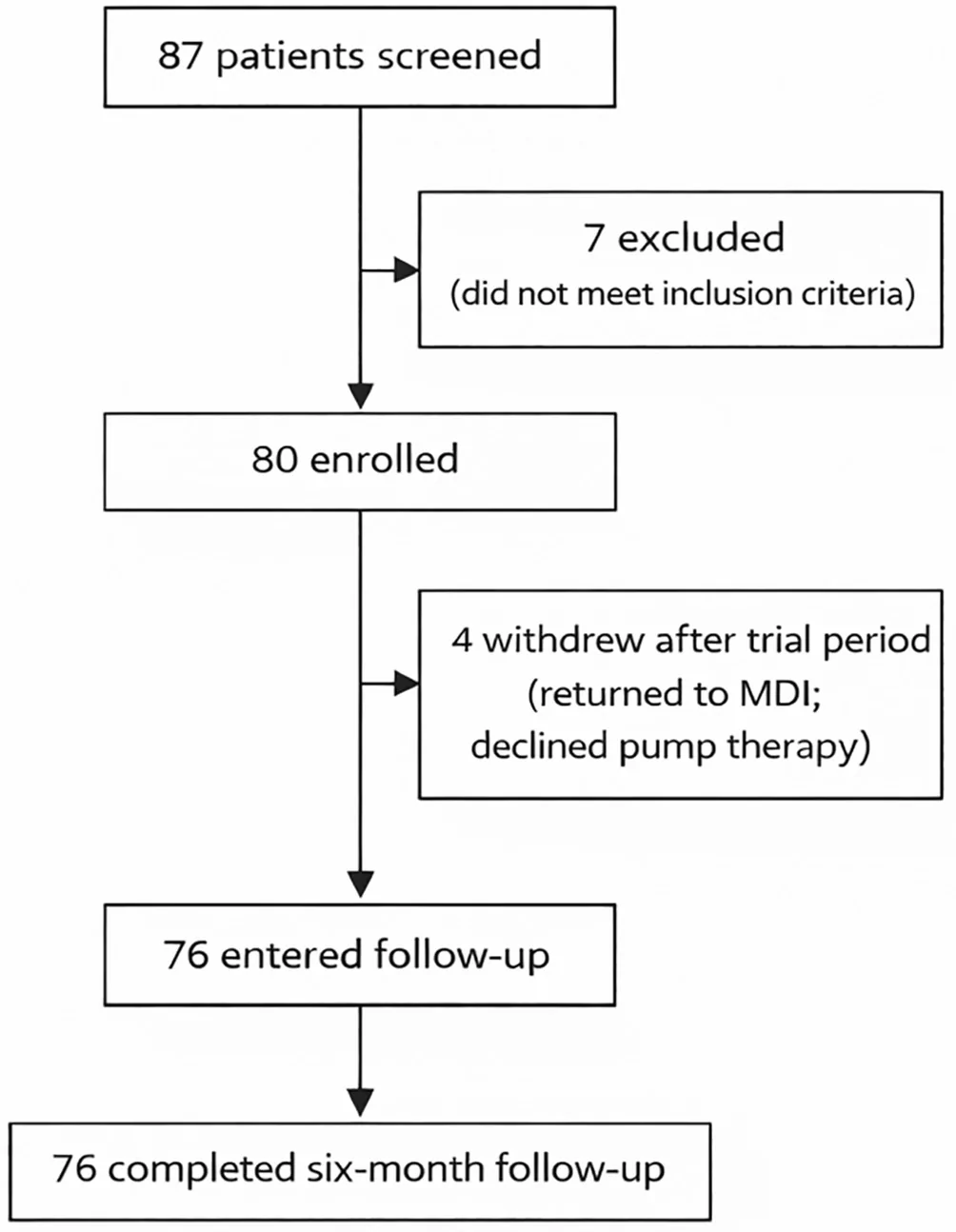
Cohort flow diagram of the study population.

Eighty‐seven patients were screened for eligibility; seven were excluded for not meeting the inclusion criteria. Of the 80 enrolled participants, four discontinued insulin pump therapy during the study period, mainly because of difficulties with device acceptance and returned to MDI. Consequently, 76 patients completed the 6‐month follow‐up and were included in the analysis. Patient selection is shown in Figure 1, and baseline clinical and demographic characteristics are reported in Table 1.

**TABLE 1:** Baseline demographic and clinical characteristics of the study population.

	Overall (*N* = 76)
Sex (Female) % (*N*)	32 (42.1%)
Age (Years) Mean ± SD min–max	13.3 ± 3.2 6–18
Diabetes duration (months) Mean ± SD	76.8 ± 52.1
Previous therapy (MDI) % (*N*)	42 (55.3%)
HbA1c baseline (%) Mean ± SD (mmol/mol)	7.4 ± 0.8 57 ± 9
BMI Z‐score baseline Mean ± SD	0.7 ± 0.9

*Note:* Data are reported as mean ± SD or *n* (%), as appropriate.

Abbreviations: BMI = body mass index; HbA1c = glycated haemoglobin; MDI = multiple daily injections.

### 3.2 TIR and TiTR

**FIGURE 2:**
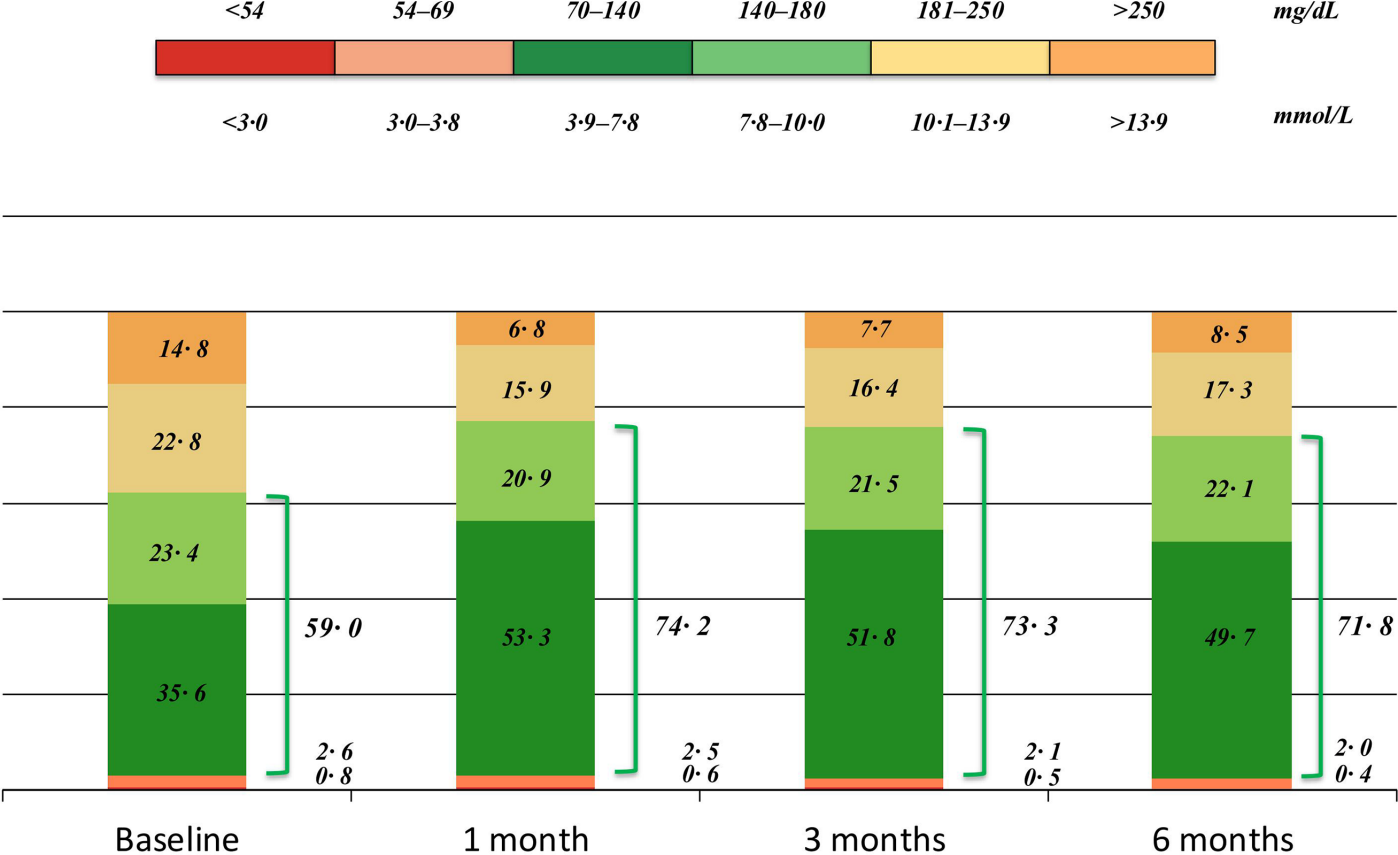
Mean percentage of time spent within predefined glucose ranges at baseline and during follow‐up visits (1, 3 and 6 months). Values are expressed as mean percentage of total continuous glucose monitoring time for each assessment period.

The distribution of time spent in predefined glycaemic ranges is reported in Table 2 and illustrated in Figure 2.

**TABLE 2:** Glycaemic control, time in glycaemic ranges, insulin doses and anthropometric measures at baseline and during follow‐up.

Colonna1	Colonna2	Colonna3	Colonna4	Colonna5
	Baseline	1 month	3 months	6 months
Glycaemic control				
HbA1c baseline (%) (mmol/mol)	7.4 ± 0.8 57 ± 9.0		6.9 ± 0.7^a^ 51 ± 8.0	6.9 ± 0.7^a^ 51 ± 8.0
Sensor glucose, mg/dL	169.4 ± 30	146.3 ± 14.1^a^	149.4 ± 16.4^a^	151.7 ± 15.4^a^
Standard deviation of sensor glucose, mg/dL	63.6 ± 16.8	58.4 ± 13.2^a^	59.1 ± 13.8^a^	59.3 ± 13.7^a^
Coefficient of variation, %	38.3 ± 6.2	39.2 ± 6.7	39.3 ± 6.2	39.3 ± 6.5
GMI, %	7.4 ± 0.7	6.8 ± 0.3^a^	6.9 ± 0.4^a^	6.9 ± 0.4^a^
Time in ranges, %				
TBR2 (< 54 mg/dL; < 3 mmol/L)	0.0 (1.0)	0.3 (0.7)	0.3 (0.7)	0.2 (0.5)
TBR1 (≥ 54 < 70 mg/dL; ≥ 3 < 3.9 mmol/L)	1.6 (2.1)	2.0 (2.4)	1.7 (2.2)	1.5 (1.6)
TBR (< 70 mg/dL; < 3.9 mmol/L)	2.0 (3.0)	2.2 (2.9)	2.0 (2.9)	1.6 (2.1)
TITR (70–140 mg/dL; 3.9–7.8 mmol/L)	32.7 (18.2)	51.5 (10.4)^a^	50.5 (12.9)^a^	49.2 (12.6)^a^
TIR (70–180 mg/dL; 3.9–10.0 mmol/L)	59.0 ± 15.6	74.2 ± 8.9^a^	73.3 ± 10.1^a^	71.8 ± 9.4^a^
TAR (> 180 mg/dL; > 10 mmol/L)	37.6 ± 16.3	22.7 ± 8.7^a^	24.2 ± 9.9^a^	25.8 ± 9.6^a^
TAR1 (> 180 ≤ 250 mg/dL; > 10 ≤ 13.9 mmol/L)	22.8 ± 7.7	15.9 ± 5.4^a^	16.4 ± 5.4^a^	17.3 ± 4.9^a^
TAR2 (> 250 mg/dL; > 13.9 mmol/L)	12.0 (17.5)	6.3 (6.3)^a^	6.5 (7.5)^a^	7.2 (7.2)^a^
Glycaemic targets				
Users reaching HbA1c ≤ 6.5%	10/76 (13.2%)		20/76 (26.3%)^a^	20/76 (26.3%)^a^
Users reaching TIR > 70%	21/76 (27.6%)	47/76 (67.1%)^a^	47/76 (61.8%)^a^	43/76 (56.6%)^a^
Users reaching TBR (< 70 mg/dL) < 4%	54/76 (71.1%)	53/76 (69.7%)	57/76 (75.0%)	54/76 (71.1%)
Users reaching TBR2 (< 54 mg/dL) < 1%	52/76 (68.4%)	62/76 (81.6%)^a^	63/76 (82.9%)^a^	67/76 (88.2%)^a^
Insulin				
Total daily dose	41.1 ± 15.5	40.1 ± 14.3	41.9 ± 15.6	41.4 ± 15.0
Basal	19.0 (10.0)	17.3 (11.2)^a^	18.6 (13.1)	18.7 (11.0)
Bolus	18.6 (15.7)	19.4 (14.5)^a^	21.2 (14.5)	21.2 (10.8)
Time with advanced hybrid closed loop active, %	0.0 (0.0)	94.0 (9.2)	96.6 (7.7)	96.4 (8.4)
Anthropometric measures				
BMI Z score	0.7 (0.9)		0.6 (1.3)	0.8 (1.4)

*Note:* Data are presented as mean (SD); variables with non‐normal distribution are also reported as median (IQR). All analyses refer to the same cohort of 76 participants evaluated at baseline, 1, 3 and 6 months. Total daily insulin dose, basal and bolus doses are expressed in units (U). BMI (body Mass Index) *Z* score was standardised for age and sex using WHO reference values.

Abbreviations: GMI = glucose management indicator; HbA1c = glycated haemoglobin; TAR = time above range; TBR = time below range; TIR = time in range; TITR = time in tight range.

^a^ Indicates *p* < 0.05 compared with baseline. Friedman test with Holm correction or repeated‐measures ANOVA with Bonferroni correction, according to the assumption of normal distribution. Paired‐samples proportion test for percentages.

TIR (70–180 mg/dL) increased from 59.0% ± 15.6% at baseline to 74.2% ± 8.9%, 73.3% ± 10.1% and 71.8% ± 9.4% at 1, 3 and 6 months, respectively (all *p* < 0.05 vs. baseline). Similarly, TiTR (70–140 mg/dL) increased from 32.7 (18.2) to 51.5 (10.4), 50.5 (12.9) and 49.2 (12.6), respectively (all *p* < 0.05 vs. baseline).

**FIGURE 3:**
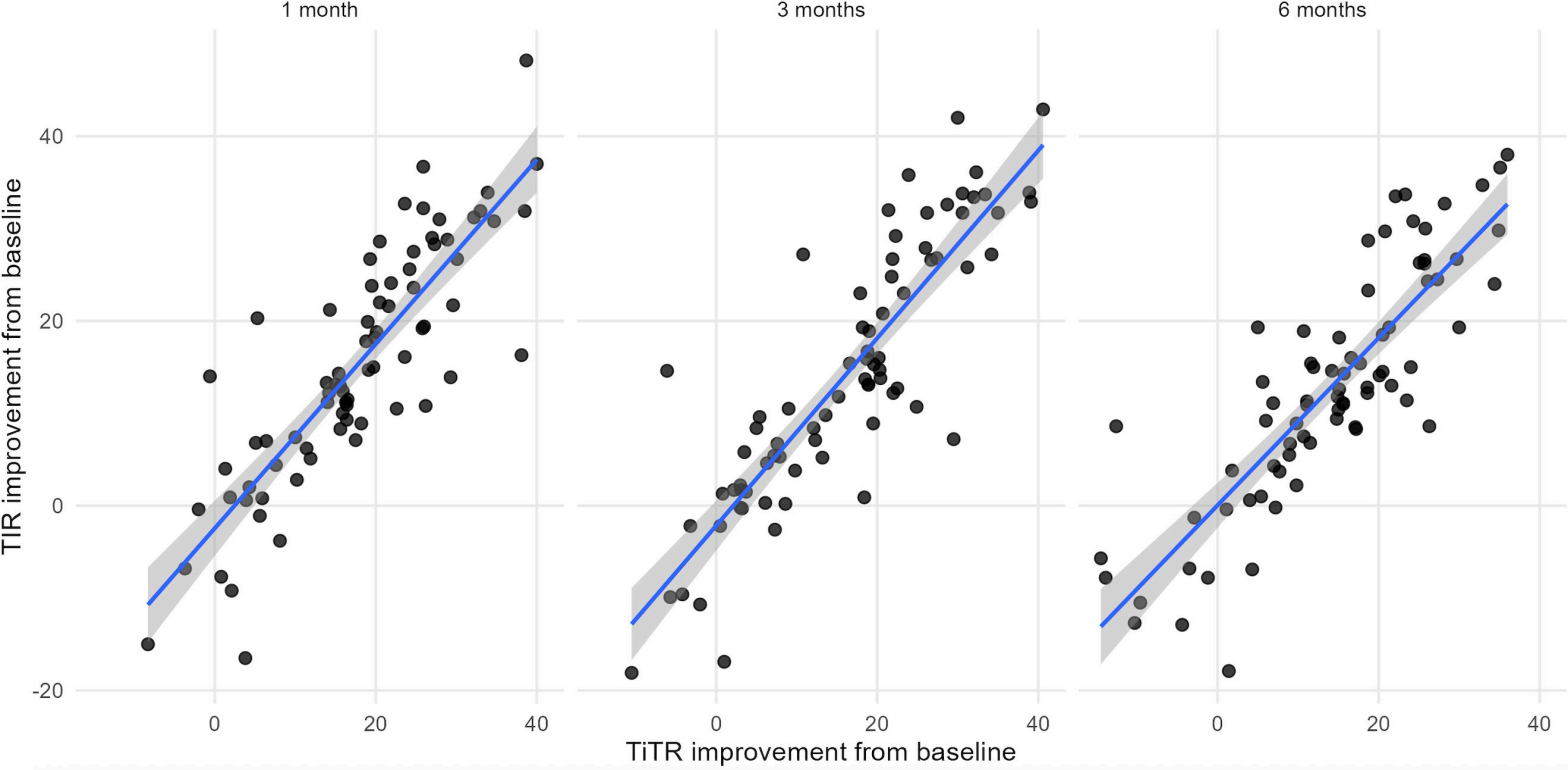
Association between improvements in Time in Tight Range (TiTR, 70–140 mg/dL) and Time in Range (TIR, 70–180 mg/dL) at 1, 3 and 6 months. Scatter plots show the relationship between changes from baseline in TiTR and TIR at each follow‐up timepoint. Pearson correlation coefficients demonstrate a strong positive association between improvements in TiTR and TIR throughout the study period (all *p* < 0.001).

As shown in Figure 3 and Table S1, changes in TiTR were strongly correlated with changes in TIR at all follow‐up visits (*r* = 0.849, 0.870, and 0.847 at 1, 3 and 6 months, respectively; all Holm‐adjusted *p* < 0.001).

Subgroup analyses showed comparable improvements in TIR and TiTR across age groups and previous treatment modalities, with no significant differences in ΔTIR or ΔTiTR between groups (Tables S1, S2, S4 and S5). In contrast, participants with baseline HbA1c ≥ 8% (*n* = 20) showed significantly greater increases in both TIR and TiTR than those with HbA1c < 8%, as demonstrated by the longitudinal analyses (Table S3) and the larger ΔTIR and ΔTiTR values (Table 3).

**TABLE 3:** Between‐group comparisons of changes according to baseline HbA1c group.

Variable	Timepoint	HbA1c < 8%	HbA1c ≥ 8%	*p*	*p* adj.
ΔHbA1c	3 months	−0.18 ± 0.70 (*n* = 56)	−1.38 ± 0.57 (*n* = 20)	< 0.001	< 0.001
ΔHbA1c	6 months	−0.35 [−0.80; −0.07] (*n* = 56)	−1.10 [−1.45; −0.50] (*n* = 20)	< 0.001	< 0.001
ΔTIR	1 month	11.54 ± 11.64 (*n* = 56)	25.46 ± 10.39 (*n* = 20)	< 0.001	< 0.001
ΔTIR	3 months	10.60 [1.45; 17.25] (*n* = 56)	29.45 [11.75; 33.82] (*n* = 20)	< 0.001	< 0.001
ΔTIR	6 months	8.99 ± 11.88 (*n* = 56)	23.52 ± 8.14 (*n* = 20)	< 0.001	< 0.001
ΔTiTR	1 month	15.01 ± 10.33 (*n* = 56)	25.20 ± 8.97 (*n* = 20)	< 0.001	< 0.001
ΔTiTR	3 months	12.87 ± 11.01 (*n* = 56)	25.50 ± 10.09 (*n* = 20)	< 0.001	< 0.001
ΔTiTR	6 months	10.88 ± 11.61 (*n* = 56)	23.08 ± 7.29 (*n* = 20)	< 0.001	< 0.001

*Note:* Patients were stratified according to baseline HbA1c using an 8% cut‐off. Delta values were calculated as follow‐up minus baseline. Values are presented as mean ± SD when both groups were normally distributed and as median [P25; P75] otherwise. The sample size used for each comparison is reported in parentheses. *p*‐values refer to between‐group comparisons. *p* adj. values were adjusted using Holm correction.

### 3.3 TAR and TBR

TAR (> 180 mg/dL), including both TAR1 (> 180 to ≤ 250 mg/dL) and TAR2 (> 250 mg/dL), decreased at all follow‐up visits compared with baseline (all *p* < 0.05 vs. baseline). In contrast, TBR (< 70 mg/dL), TBR1 (≥ 54 to < 70 mg/dL) and TBR2 (< 54 mg/dL) remained unchanged throughout follow‐up. Similar reductions in hyperglycaemic exposure and stable hypoglycaemia‐related metrics were observed across baseline HbA1c categories, age groups and previous treatment modalities (Tables [Link], [Link], [Link]).

### 3.4 Glycaemic Variability and GMI

Mean sensor glucose decreased from 169.4 ± 30.0 mg/dL at baseline to 146.3 ± 14.1 mg/dL at 1 month, 149.4 ± 16.4 mg/dL at 3 months and 151.7 ± 15.4 mg/dL at 6 months (all *p* < 0.05 vs. baseline). Glucose variability, expressed as the standard deviation of sensor glucose, was also lower at follow‐up compared with baseline (63.6 ± 16.8 mg/dL), reaching 58.4 ± 13.2, 59.1 ± 13.8 and 59.3 ± 13.7 mg/dL at 1, 3 and 6 months, respectively (all *p* < 0.05 vs. baseline). The coefficient of variation remained stable throughout the study period.

GMI improved after 1 month (7.4% ± 0.7% vs. 6.8% ± 0.3%) and remained lower than baseline at both 3 and 6 months (6.9% ± 0.4%; all *p* < 0.05 vs. baseline).

Similar improvements were observed across baseline HbA1c categories, age groups and previous treatment modalities (Tables S2, S3 and S4). Reductions in the standard deviation of sensor glucose were most evident amongst participants with baseline HbA1c ≥ 8% and those previously treated with MDI, whereas the coefficient of variation remained largely unchanged across all analysed subgroups.

### 3.5 HbA1c, Glycaemic Targets

HbA1c, assessed at baseline and at 3 and 6 months, decreased from 7.4% ± 0.8% (57 ± 9.0 mmol/mol) to 6.9% ± 0.7% (51 ± 8.0 mmol/mol) at both follow‐up visits (*p* < 0.05 vs. baseline).

The proportion of participants achieving predefined glycaemic targets is reported in Table 2. Achievement of HbA1c ≤ 6.5% increased from 13.2% at baseline to 26.3% at both follow‐up visits, whilst achievement of TIR > 70% increased from 27.6% at baseline to 67.1%, 61.8% and 56.6% at 1, 3 and 6 months, respectively.

HbA1c improved across all analysed subgroups. However, participants with baseline HbA1c ≥ 8% (*n* = 20) showed significantly greater reductions than those with HbA1c < 8%, as demonstrated by both the longitudinal analyses (Table S3) and the between‐group comparisons of ΔHbA1c (Table 3). Achievement of HbA1c and TIR targets was more frequent amongst participants with baseline HbA1c < 8% and those previously treated with MDI, whereas comparable benefits were observed across age groups (Tables [Link], [Link], [Link]).

### 3.6 Basal/Bolus Insulin Changes

Total daily insulin dose remained stable throughout follow‐up. Basal insulin dose decreased at 1 month compared with baseline (19.0 [10.0] vs. 17.3 [11.2]; *p* < 0.05), whereas values at 3 and 6 months were not significantly different. Conversely, bolus insulin dose increased at 1 month (18.6 [15.7] vs. 19.4 [14.5]; *p* < 0.05) and remained numerically higher thereafter without reaching statistical significance (Table 2).

Similar patterns of basal and bolus insulin distribution were observed across baseline HbA1c categories, age groups and previous treatment modalities, although the magnitude of change varied amongst subgroups (Tables [Link], [Link], [Link]).

### 3.7 Automode Use and Anthropometric Measures

Auto Mode use remained consistently high throughout follow‐up, reaching 94.0% (9.2), 96.6% (7.7) and 96.4% (8.4) at 1, 3 and 6 months, respectively (Table 2). BMI z‐score remained stable from baseline (0.7 [0.9]) to 3 months (0.6 [1.3]) and 6 months (0.8 [1.4]). High Auto Mode use was consistently maintained across baseline HbA1c categories, age groups and previous treatment modalities. Although a modest increase in BMI *z*‐score was observed amongst participants with baseline HbA1c ≥ 8%, no evidence of clinically relevant weight gain was detected during follow‐up (Tables [Link], [Link], [Link]). No episodes of severe hypoglycaemia or DKA occurred during follow‐up.

## 4 Discussion

This study represents the first real‐world clinical evaluation of the A8 TouchCare Nano AID system, specifically focusing on the performance of the APGO algorithm in a paediatric population. Our findings demonstrate that the switch to this AID system leads to a rapid and sustained improvement in glycaemic control, as evidenced by the significant increase in TIR and the reduction in HbA1c and GMI levels observed as early as 1 month after initiation and maintained throughout the 6‐month follow‐up.

The improvement in glycaemic control observed in our cohort was further supported by the marked increase in TiTR (70–140 mg/dL) and the significant reduction in mean sensor glucose levels. These benefits were achieved without increasing hypoglycaemia risk, as TBR remained stable throughout follow‐up, with no episodes of severe hypoglycaemia or DKA. Notably, improvements in TiTR were strongly correlated with improvements in TIR at all follow‐up timepoints, indicating that better overall glycaemic control was accompanied by increased time spent within a near‐normoglycaemic range. These findings support the clinical relevance of TiTR as a complementary outcome measure in paediatric AID studies.

Subgroup analyses demonstrated consistent benefits across age groups and previous treatment modalities. As reported with other AID systems, participants with baseline HbA1c ≥ 8% experienced the greatest improvements in HbA1c, TIR and TiTR, likely reflecting a greater opportunity for metabolic optimization.

In line with current ISPAD recommendations, an increasing proportion of participants achieved recommended glycaemic targets following AID initiation whilst maintaining low exposure to hypoglycaemia throughout follow‐up. This finding is particularly relevant given that ISPAD recommends an HbA1c target ≤ 6.5% only when it can be achieved safely, ideally with the support of CGM and AID technologies [13]. Nevertheless, recent meta‐analytic evidence [14] indicates that, despite significant reductions in HbA1c, attainment of stringent glycaemic targets remains challenging for some individuals, particularly adolescents and those with higher baseline HbA1c levels. Consistent with these observations, our results suggest that AID therapy can facilitate achievement of recommended targets in a substantial proportion of children and adolescents without compromising safety.

However, AID technology alone does not fully account for these outcomes. Participants were managed within hospital tertiary paediatric diabetes centres equipped with multidisciplinary diabetes teams providing structured ongoing and individualised education, psychological assessment, advanced carbohydrate‐counting training and dedicated technical support. Furthermore, all participating centres provided a 24‐h, 7‐day‐a‐week on‐call paediatric diabetology service, ensuring immediate access to specialist support for urgent clinical issues, acute complications, insulin dose adjustments and diabetes‐related emergencies. This continuous availability of expert diabetes care represents a distinctive feature of tertiary centres and may contribute to the high levels of treatment adherence and favourable clinical outcomes observed in this cohort [15].

A key highlight of our study is the remarkably high percentage of time spent in Auto Mode, which remained above 96% at both the third and sixth month. The slightly lower value observed at 1 month reflects the predefined run‐in period in PLGS mode following device initiation. This level of persistence is amongst the highest reported in the literature for paediatric cohorts [16] and suggests that the system is not only effective but also highly acceptable.

Although patch pumps are generally well accepted by patients, concerns have been raised regarding the environmental impact associated with the large amount of disposable material generated by these systems [17, 18]. Nevertheless, the A8 TouchCare Nano system incorporates reusable components that could partially mitigate the environmental impact typically associated with disposable patch pump technologies.

No significant changes in total daily insulin dose were observed over the 6‐month follow‐up period. At 1‐month, basal insulin requirements decreased significantly, accompanied by a corresponding increase in bolus insulin delivery; however, from 3 to 6 months, differences in basal and bolus doses were no longer statistically significant. These findings are consistent with previous evidence demonstrating improved insulin efficiency with continuous subcutaneous insulin infusion (CSII) compared with MDI [19]. Notably, glycaemic control improved substantially despite the absence of changes in total daily insulin dose. Although not statistically significant, a modest predominance of bolus over basal insulin delivery persisted during follow‐up. This pattern may reflect the intrinsic characteristics of the APGO algorithm, which permits up to 30 corrective boluses per hour.

No significant changes in BMI *z*‐score were observed in the overall cohort over the 6‐month follow‐up period. Although a modest increase in BMI *z*‐score was observed amongst participants with baseline HbA1c ≥ 8%, no clinically relevant weight gain was detected during follow‐up. Our findings are consistent with previous reports in paediatric populations using automated insulin delivery systems [20]. The relatively short duration of observation precludes definitive conclusions regarding long‐term anthropometric trajectories.

Several limitations of this study must also be considered. The observational design and absence of a parallel control group preclude causal inference and limit conclusions regarding comparative effectiveness versus alternative therapeutic strategies. Although the magnitude of improvement was substantial, residual confounding related to structured education, multi‐disciplinary care and centre‐specific expertise cannot be excluded. The highly specialised nature of the participating centres may also limit generalizability to less structured clinical settings.

In addition, the relatively modest sample size reduces the precision of effect estimates and may limit broader generalizability. Given that the system is relatively new and not yet widely adopted, the number of eligible participants was inherently limited. Larger, multicentre studies with longer follow‐up are warranted to confirm these findings and to further define the long‐term clinical impact of this technology in diverse real‐world settings.

Another limitation is that participants used different CGM systems before study entry and the potential influence of baseline sensor heterogeneity on the observed outcomes cannot therefore be completely excluded.

The A8 TouchCare System has been subject to regulatory restrictions in some European countries [21, 22, 23], mainly related to concerns regarding CGM accuracy. However, no safety notices or commercialization restrictions were issued in Italy during the study period. Although previous real‐world evaluations reported some concerns regarding sensor performance, most users expressed overall satisfaction and elected to continue using the system [11]. In this study by Borrella et al., despite the relatively small sample size and short observation period, and although some concerns regarding sensor accuracy were reported, most participants expressed overall satisfaction with the system and chose to continue using it after study completion.

Other commercially available CGM sensors and AID systems have also been subject to European safety notices and field safety notifications. In addition, findings from clinical studies and review articles have reported issues related to sensor accuracy and device malfunctions involving several widely used systems [24]. One possible explanation is that critical appraisal of the available evidence has shown that several CGM devices received CE marking for broad clinical indications extending beyond the level of evidence typically required by other international regulatory authorities [25].

Studies such as ours contribute to expanding the evidence base for less extensively studied AID systems, helping clinicians and regulatory authorities to better understand their safety profile and real‐world performance. As shown in Table 2, CGM‐derived metrics, particularly GMI values, were closely consistent with the observed HbA1c results throughout follow‐up, indirectly supporting the overall reliability of sensor‐derived glycaemic estimates in our cohort and the safety of system use within specialised paediatric diabetes centres with structured and continuous therapeutic education and close clinical follow‐up.

Further multi‐centre studies with larger sample sizes and longer follow‐up are warranted to confirm these findings and to better define the long‐term effectiveness, safety and real‐world applicability of this AID system in paediatric populations.

## Author Contributions

A.Z. and B.P. codesigned the study. A.Z., G.I., A.S.R., A.C., A.G.F. and A.F. provided patient care and collected clinical data. A.Z. performed the statistical analyses. A.Z. drafted the manuscript. D.I., E.M.D.G., S.T. and B.P. supervised the study and contributed to data interpretation. All authors critically reviewed the manuscript and contributed to the interpretation of the results. A.Z. takes responsibility for the integrity of the data and the accuracy of the data analysis. All authors had access to the data, reviewed the paper before publication and approved the final version for submission.

## Funding

The authors have nothing to report.

## Ethics Statement

Participants and their caregivers have provided written informed consent.

## Conflicts of Interest

The authors declare no conflicts of interest.

## Supporting information


**Table S1:** Correlation between improvements in Time in Tight Range (TiTR, 70–140 mg/dL) and Time in Range (TIR, 70–180 mg/dL) from baseline to 1, 3 and 6 months. Pearson correlation analyses were performed to assess the association between changes in TiTR and changes in TIR at each follow‐up timepoint. Improvements were calculated as the difference between follow‐up and baseline values. *p*‐values were adjusted for multiple comparisons using the Holm correction method.

**Table S2:** Glycaemic Outcomes According to Previous Therapy: SAP (*N* = 34).

**Table S3:** Longitudinal glycaemic, insulin, and anthropometric outcomes according to baseline HbA1c category. Panel A includes participants with baseline HbA1c < 8% (*n* = 56), and Panel B includes participants with baseline HbA1c ≥ 8% (*n* = 20). Data are presented as mean ± SD for normally distributed variables and median [P25; P75] for non‐normally distributed variables. Categorical outcomes are presented as *n*/*N* (%). Global *p*‐values refer to repeated‐measures ANOVA or Friedman test, as appropriate. When the global test was significant, post‐hoc comparisons versus baseline were performed using Holm correction. * indicates *p* < 0.05 versus baseline.

**Table S4:** Longitudinal glycaemic, insulin, and anthropometric outcomes according to age group. Panel A includes participants aged ≥ 12 years (*n* = 49), and Panel B includes participants aged < 12 years (*n* = 27). Data are presented as mean ± SD for normally distributed variables and median [P25; P75] for non‐normally distributed variables. Categorical outcomes are presented as *n*/*N* (%). Global *p*‐values refer to repeated‐measures ANOVA or Friedman test, as appropriate. When the global test was significant, post‐hoc comparisons versus baseline were performed using Holm correction. * indicates *p* < 0.05 versus baseline.

**Table S5:** Between‐group comparisons of changes in HbA1c, Time in Range (TIR, 70–180 mg/dL) and Time in Tight Range (TiTR, 70–140 mg/dL) according to age group (< 12 years vs. ≥ 12 years). Delta (Δ) values were calculated as follow‐up minus baseline. Values are presented as mean ± SD for normally distributed variables and median [P25; P75] for non‐normally distributed variables. *p*‐values refer to between‐group comparisons, and adjusted *p*‐values were calculated using the Holm correction method. Sample size for each comparison is reported in parentheses. No significant differences in ΔHbA1c, ΔTIR or ΔTiTR were observed between age groups at any follow‐up timepoint.

**Table S6:** Between‐group comparisons of changes in HbA1c, Time in Range (TIR, 70–180 mg/dL) and Time in Tight Range (TiTR, 70–140 mg/dL) according to previous treatment modality (multiple daily injections [MDI] vs. sensor‐augmented pump therapy [SAP]). Delta (Δ) values were calculated as follow‐up minus baseline. Values are presented as mean ± SD for normally distributed variables and median [P25; P75] for non‐normally distributed variables. *p*‐values refer to between‐group comparisons, and adjusted *p*‐values were calculated using the Holm correction method. Sample size for each comparison is reported in parentheses. No significant differences in ΔHbA1c, ΔTIR or ΔTiTR were observed between treatment groups at any follow‐up timepoint.

## Data Availability

Deidentified individual participant data that underlie the results reported in this article, together with a data dictionary defining each variable, will be made available upon reasonable request to the corresponding author. Additional study‐related documents, including the study protocol and statistical analysis plan, will also be available upon request. Data will be available beginning with publication and without an end date. Data will be shared with qualified researchers for purposes of replicating procedures and results or for other analyses approved by the study investigators, following submission of a methodologically sound proposal and signing of a data access agreement. No participant‐identifiable data will be shared.
