# Supplementary material for: Real‐World Effectiveness and Safety of a Tubeless Automated Insulin Delivery System With an Adaptive Control Algorithm (APGO) in Children and Adolescents With Type 1 Diabetes: A Multicentre Observational Study
Source: Diabetes Obes Metab. 2026 Jul 23;28(10):9275–83. doi: 10.1111/dom.71101 (PMC13538724; doi:10.1111/dom.71101)
Supplement: Supplementary file 1 [file DOM-28-9275-s001.docx]

Supplementary Table 2A

*Glycaemic Outcomes According to Previous Therapy: SAP (N=34)*

| **Variable** | **Baseline** | **1 month** | **3 months** | **6 months** | **p** |
| --- | --- | --- | --- | --- | --- |
| ***Glycaemic control*** |  |  |  |  |  |
| HbA1c (%) | 7.3 [7.0; 7.9] |  | 7.0 [6.7; 7.3]* | 7.0 [6.5; 7.3]* | 0.009 |
| Sensor glucose, mg/dL | 172.0 [151.2; 189.0] | 152.0 [143.8; 158.8]* | 154.5 [144.2; 161.8]* | 155.5 [144.2; 163.0]* | < 0.001 |
| Standard deviation of sensor glucose, mg/dL | 66.9 ± 16.4 | 62.5 ± 9.3 | 63.6 ± 12.2 | 62.6 ± 12.9 | 0.290 |
| Coefficient of variation, % | 40.0 [34.0; 43.0] | 40.5 [38.0; 43.0] | 40.0 [38.0; 44.0] | 40.0 [38.2; 44.8] | 0.530 |
| GMI, % | 7.5 ± 0.6 | 6.9 ± 0.3* | 7.0 ± 0.4* | 7.0 ± 0.4* | < 0.001 |
| ***Time in ranges, %*** |  |  |  |  |  |
| TBR2 (<54 mg/dL; <3 mmol/L) | 0.4 [0.0; 1.0] | 0.3 [0.1; 0.8] | 0.4 [0.1; 0.9] | 0.3 [0.1; 0.8] | 0.629 |
| TBR1 (>=54 <70 mg/dL; >=3 <3.9 mmol/L) | 1.9 [0.8; 3.5] | 2.1 [1.4; 3.3] | 1.9 [1.0; 3.2] | 1.8 [1.0; 2.6] | 0.898 |
| TBR (<70 mg/dL; <3.9 mmol/L) | 2.0 [1.0; 4.9] | 2.2 [1.5; 4.4] | 2.3 [1.1; 4.2] | 2.1 [1.2; 3.3] | 0.970 |
| TITR (70-140 mg/dL; 3.9-7.8 mmol/L) | 34.2 ± 9.5 | 49.4 ± 6.7* | 47.8 ± 8.7* | 48.5 ± 7.6* | <0 .001 |
| TIR (70-180 mg/dL; 3.9-10.0 mmol/L) | 57.9 ± 12.5 | 70.6 ± 7.3* | 69.5 ± 9.7* | 70.1 ± 7.9* | < 0.001 |
| TAR (>180 mg/dL; >10 mmol/L) | 38.8 ± 13.5 | 26.2 ± 7.2* | 27.9 ± 9.5* | 27.3 ± 8.2* | < 0.001 |
| TAR1 (>180 <=250 mg/dL; >10 <=13.9 mmol/L) | 23.6 ± 6.8 | 17.8 ± 4.2* | 18.4 ± 4.8* | 18.4 ± 4.3* | < 0.001 |
| TAR2 (>250 mg/dL; >13.9 mmol/L) | 14.8 [5.9; 22.9] | 6.9 [5.5; 10.8]* | 8.8 [5.5; 10.9]* | 8.2 [4.8; 10.7]* | 0.001 |
| ***Glycaemic targets*** |  |  |  |  |  |
| Users reaching HbA1c <=6.5% | 5/34 (14.7%) |  | 5/34 (14.7%) | 10/33 (30.3%) | 0.146 |
| Users reaching TIR >70% | 9/34 (26.5%) | 20/34 (58.8%)* | 16/34 (47.1%) | 17/34 (50.0%) | 0.019 |
| Users reaching TBR (<70 mg/dL) <4% | 23/34 (67.6%) | 22/34 (64.7%) | 23/34 (67.6%) | 26/34 (76.5%) | 0.672 |
| Users reaching TBR2 (<54 mg/dL) <1% | 20/34 (58.8%) | 26/34 (76.5%) | 26/34 (76.5%) | 29/34 (85.3%) | 0.054 |
| ***Insulin*** |  |  |  |  |  |
| Total daily dose | 43.1 ± 12.0 | 43.7 ± 11.1 | 47.8 ± 12.1* | 46.8 ± 11.9 | 0.004 |
| Basal | 19.2 [18.0; 22.6] | 21.1 [16.0; 25.7] | 23.2 [17.7; 25.8] | 20.8 [17.3; 27.6] | 0.057 |
| Bolus | 18.6 [14.4; 29.8] | 19.5 [15.7; 28.7] | 23.0 [19.1; 31.8]* | 22.3 [19.9; 30.0]* | 0.011 |
| Time with advanced hybrid closed loop active, % |  | 95.5 [87.1; 99.6] | 99.1 [93.6; 99.8] | 99.5 [93.6; 99.8] | 0.249 |
| ***Anthropometric measures*** |  |  |  |  |  |
| BMI Z score | 0.5 ± 0.8 |  | 0.5 ± 0.7 | 0.5 ± 0.8 | 0.926 |

Supplementary Table 2B

*Glycaemic Outcomes According to Previous Therapy: MDI (N=42)*

| **Variable** | **Baseline** | **1 month** | **3 months** | **6 months** | **p** |
| --- | --- | --- | --- | --- | --- |
| ***Glycaemic control*** |  |  |  |  |  |
| HbA1c (%) | 7.2 [6.8; 8.1] |  | 6.8 [6.3; 7.1]* | 6.8 [6.4; 7.1]* | < 0.001 |
| Sensor glucose, mg/dL | 166.2 ± 34.4 | 141.9 ± 14.8* | 144.9 ± 16.0* | 148.8 ± 14.7* | < 0.001 |
| Standard deviation of sensor glucose, mg/dL | 61.0 ± 16.8 | 55.0 ± 14.9 | 55.5 ± 14.0 | 56.7 ± 13.9 | 0.021 |
| Coefficient of variation, % | 37.4 ± 5.5 | 37.7 ± 7.8 | 38.1 ± 6.9 | 38.0 ± 6.8 | 0.815 |
| GMI, % | 7.3 ± 0.8 | 6.7 ± 0.4* | 6.8 ± 0.4* | 6.9 ± 0.3* | < 0.001 |
| ***Time in ranges, %*** |  |  |  |  |  |
| TBR2 (<54 mg/dL; <3 mmol/L) | 0.0 [0.0; 0.2] | 0.2 [0.0; 0.5] | 0.1 [0.0; 0.6] | 0.2 [0.0; 0.4] | 0.016 |
| TBR1 (>=54 <70 mg/dL; >=3 <3.9 mmol/L) | 1.0 [1.0; 3.0] | 1.9 [1.2; 3.8] | 1.4 [0.8; 3.0] | 1.2 [0.7; 2.5] | 0.020 |
| TBR (<70 mg/dL; <3.9 mmol/L) | 1.6 [1.0; 3.8] | 2.1 [1.3; 4.2] | 1.8 [0.9; 3.5] | 1.3 [0.7; 3.0] | 0.035 |
| TITR (70-140 mg/dL; 3.9-7.8 mmol/L) | 32.1 [23.2; 51.1] | 53.5 [49.4; 62.1]* | 53.7 [47.5; 62.1]* | 50.0 [44.5; 56.8]* | < 0.001 |
| TIR (70-180 mg/dL; 3.9-10.0 mmol/L) | 59.9 ± 17.8 | 77.1 ± 9.2* | 76.4 ± 9.4* | 73.2 ± 10.4* | < 0.001 |
| TAR (>180 mg/dL; >10 mmol/L) | 36.6 ± 18.4 | 19.9 ± 8.9* | 21.2 ± 9.3* | 24.6 ± 10.6* | < 0.001 |
| TAR1 (>180 <=250 mg/dL; >10 <=13.9 mmol/L) | 24.0 [17.0; 29.0] | 15.1 [11.7; 17.8]* | 14.0 [11.1; 19.3]* | 16.8 [13.8; 20.1]* | < 0.001 |
| TAR2 (>250 mg/dL; >13.9 mmol/L) | 11.2 [4.1; 22.0] | 5.3 [2.0; 8.3]* | 5.0 [2.1; 11.3]* | 5.5 [3.2; 11.0]* | < 0.001 |
| ***Glycaemic targets*** |  |  |  |  |  |
| Users reaching HbA1c <=6.5% | 5/42 (11.9%) |  | 12/42 (28.6%) | 17/42 (40.5%)* | 0.002 |
| Users reaching TIR >70% | 12/42 (28.6%) | 31/42 (73.8%)* | 31/42 (73.8%)* | 26/42 (61.9%)* | < 0.001 |
| Users reaching TBR (<70 mg/dL) <4% | 31/42 (73.8%) | 31/42 (73.8%) | 34/42 (81.0%) | 36/42 (85.7%) | 0.229 |
| Users reaching TBR2 (<54 mg/dL) <1% | 32/42 (76.2%) | 36/42 (85.7%) | 37/42 (88.1%) | 38/42 (90.5%) | 0.137 |
| ***Insulin*** |  |  |  |  |  |
| Total daily dose | 37.3 [24.5; 54.0] | 34.6 [24.8; 45.5] | 32.7 [21.9; 47.2] | 34.2 [25.4; 48.0] | 0.815 |
| Basal | 18.3 [13.0; 26.0] | 14.1 [10.8; 20.1]* | 14.9 [10.6; 21.2]* | 14.2 [11.6; 19.9]* | < 0.001 |
| Bolus | 18.3 [11.0; 27.5] | 19.8 [13.4; 28.5]* | 19.4 [13.2; 27.5] | 18.4 [13.6; 25.2] | 0.011 |
| Time with advanced hybrid closed loop active, % |  | 99.6 [96.1; 100.0] | 99.7 [98.2; 100.0] | 99.7 [99.0; 100.0] | .320 |
| ***Anthropometric measures*** |  |  |  |  |  |
| BMI Z score | 0.8 [0.2; 1.5] |  | 0.9 [0.1; 1.7] | 0.9 [0.1; 1.6] | 0.626 |

**Supplementary Table S2** Longitudinal glycaemic, insulin, and anthropometric outcomes according to previous treatment modality. Panel A includes participants previously treated with sensor-augmented pump therapy (SAP; n = 34), and Panel B includes participants previously treated with multiple daily injections (MDI; n = 42). Data are presented as mean ± SD for normally distributed variables and median [P25; P75] for non-normally distributed variables. Categorical outcomes are presented as n/N (%). Global p-values refer to repeated-measures ANOVA or Friedman test, as appropriate. When the global test was significant, post-hoc comparisons versus baseline were performed using Holm correction. * indicates p < 0.05 versus baseline.
