# Supplementary material for: Real‐World Effectiveness and Safety of a Tubeless Automated Insulin Delivery System With an Adaptive Control Algorithm (APGO) in Children and Adolescents With Type 1 Diabetes: A Multicentre Observational Study
Source: Diabetes Obes Metab. 2026 Jul 23;28(10):9275–83. doi: 10.1111/dom.71101 (PMC13538724; doi:10.1111/dom.71101)
Supplement: Supplementary file 2 [file DOM-28-9275-s002.docx]

Supplementary Table 3A

*Glycaemic Outcomes According to Baseline HbA1c Group: HbA1c <8% (N=56)*

| **Variable** | **Baseline** | **1 month** | **3 months** | **6 months** | **p** |
| --- | --- | --- | --- | --- | --- |
| ***Glycaemic control*** |  |  |  |  |  |
| HbA1c (%) | 7.1 [6.8; 7.5] |  | 6.9 [6.5; 7.2]* | 6.6 [6.4; 7.1]* | < 0.001 |
| Sensor glucose, mg/dL | 160.2 ± 25.0 | 144.4 ± 14.2* | 147.8 ± 15.5* | 150.2 ± 16.1* | < 0.001 |
| Standard deviation of sensor glucose, mg/dL | 58.0 [49.0; 66.5] | 57.0 [48.0; 63.0] | 58.0 [46.8; 65.2] | 54.0 [47.8; 65.2] | 0.270 |
| Coefficient of variation, % | 37.8 ± 6.4 | 38.1 ± 6.3 | 38.0 ± 5.6 | 38.1 ± 6.4 | 0.980 |
| GMI, % | 7.2 ± 0.6 | 6.8 ± 0.3* | 6.8 ± 0.4* | 6.9 ± 0.4* | < 0.001 |
| ***Time in ranges, %*** |  |  |  |  |  |
| TBR2 (<54 mg/dL; <3 mmol/L) | 0.0 [0.0; 1.0] | 0.2 [0.0; 0.6] | 0.2 [0.1; 0.6] | 0.2 [0.0; 0.5] | 0.190 |
| TBR1 (>=54 <70 mg/dL; >=3 <3.9 mmol/L) | 1.8 [0.7; 3.0] | 2.0 [1.2; 3.3] | 1.8 [0.9; 2.6] | 1.5 [0.9; 2.5] | 0.238 |
| TBR (<70 mg/dL; <3.9 mmol/L) | 2.0 [1.0; 4.2] | 2.2 [1.3; 4.1] | 2.0 [1.0; 3.2] | 1.6 [0.9; 3.1] | 0.342 |
| TITR (70-140 mg/dL; 3.9-7.8 mmol/L) | 38.5 [29.8; 49.5] | 53.2 [48.0; 58.5]* | 50.8 [46.3; 58.8]* | 49.8 [43.5; 57.2]* | < 0.001 |
| TIR (70-180 mg/dL; 3.9-10.0 mmol/L) | 64.2 ± 14.2 | 75.8 ± 9.0* | 74.7 ± 9.6* | 73.2 ± 9.6* | < 0.001 |
| TAR (>180 mg/dL; >10 mmol/L) | 32.6 ± 14.8 | 21.4 ± 8.7* | 23.0 ± 9.5* | 24.6 ± 9.6* | < 0.001 |
| TAR1 (>180 <=250 mg/dL; >10 <=13.9 mmol/L) | 21.4 ± 7.9 | 15.4 ± 5.5* | 16.2 ± 5.4* | 16.9 ± 5.2* | < 0.001 |
| TAR2 (>250 mg/dL; >13.9 mmol/L) | 8.9 [4.4; 20.0] | 5.7 [3.0; 8.5]* | 5.8 [3.0; 10.1]* | 5.8 [3.1; 10.3]* | < 0.001 |
| ***Glycaemic targets*** |  |  |  |  |  |
| Users reaching HbA1c <=6.5% | 10/56 (17.9%) |  | 15/56 (26.8%) | 25/56 (44.6%)* | 0.002 |
| Users reaching TIR >70% | 21/56 (37.5%) | 42/56 (75.0%)* | 37/56 (66.1%)* | 35/56 (62.5%)* | < 0.001 |
| Users reaching TBR (<70 mg/dL) <4% | 40/56 (71.4%) | 40/56 (71.4%) | 46/56 (82.1%) | 46/56 (82.1%) | 0.179 |
| Users reaching TBR2 (<54 mg/dL) <1% | 39/56 (69.6%) | 48/56 (85.7%) | 48/56 (85.7%) | 51/56 (91.1%)* | 0.006 |
| ***Insulin*** |  |  |  |  |  |
| Total daily dose | 39.6 ± 14.5 | 38.3 ± 12.7 | 39.1 ± 13.9 | 39.6 ± 14.3 | 0.556 |
| Basal | 20.0 ± 7.6 | 17.6 ± 7.4* | 17.9 ± 7.7* | 18.3 ± 7.9* | 0.001 |
| Bolus | 16.0 [12.6; 28.0] | 18.5 [14.0; 27.0]* | 20.4 [14.2; 25.5] | 20.3 [15.8; 27.3]* | 0.010 |
| Time with advanced hybrid closed loop active, % |  | 99.3 [93.7; 99.8] | 99.5 [96.9; 99.9] | 99.7 [97.9; 99.9] | 0.430 |
| ***Anthropometric measures*** |  |  |  |  |  |
| BMI Z score | 0.7 ± 0.8 |  | 0.6 ± 0.9 | 0.6 ± 0.9 | 0.333 |

Supplementary Table 3B

*Glycaemic Outcomes According to Baseline HbA1c Group: HbA1c >=8% (N=20)*

| **Variable** | **Baseline** | **1 month** | **3 months** | **6 months** | **p** |
| --- | --- | --- | --- | --- | --- |
| ***Glycaemic control*** |  |  |  |  |  |
| HbA1c (%) | 8.3 [8.2; 8.4] |  | 6.9 [6.7; 7.4]* | 7.3 [6.8; 7.8]* | < 0.001 |
| Sensor glucose, mg/dL | 197.0 [187.5; 203.5] | 153.0 [138.0; 160.5]* | 154.5 [141.8; 163.2]* | 158.5 [148.0; 163.8]* | <0 .001 |
| Standard deviation of sensor glucose, mg/dL | 79.5 [74.2; 83.5] | 65.5 [60.2; 71.2]* | 66.0 [56.8; 74.8]* | 65.0 [56.8; 78.0]* | 0.003 |
| Coefficient of variation, % | 39.8 ± 5.2 | 42.3 ± 7.0 | 42.9 ± 6.5 | 42.5 ± 6.0 | 0.058 |
| GMI, % | 8.1 [7.8; 8.2] | 7.0 [6.8; 7.1]* | 7.0 [6.7; 7.2]* | 7.1 [6.9; 7.2]* | < 0.001 |
| ***Time in ranges, %*** |  |  |  |  |  |
| TBR2 (<54 mg/dL; <3 mmol/L) | 0.3 [0.0; 1.0] | 0.3 [0.2; 1.1] | 0.6 [0.1; 0.9] | 0.2 [0.1; 0.5] | 0.101 |
| TBR1 (>=54 <70 mg/dL; >=3 <3.9 mmol/L) | 1.4 [1.0; 3.0] | 1.8 [1.4; 4.4] | 2.0 [0.8; 3.8] | 1.6 [1.0; 2.5] | 0.351 |
| TBR (<70 mg/dL; <3.9 mmol/L) | 1.9 [1.0; 4.0] | 2.3 [1.5; 5.8] | 3.0 [1.0; 4.9] | 1.9 [1.1; 2.7] | 0.313 |
| TITR (70-140 mg/dL; 3.9-7.8 mmol/L) | 24.4 [20.6; 27.2] | 50.1 [43.8; 51.8]* | 49.4 [43.9; 55.2]* | 47.5 [43.5; 50.6]* | < 0.001 |
| TIR (70-180 mg/dL; 3.9-10.0 mmol/L) | 44.4 ± 8.5 | 69.8 ± 7.5* | 69.4 ± 10.6* | 67.9 ± 7.9* | < 0.001 |
| TAR (>180 mg/dL; >10 mmol/L) | 51.6 ± 11.7 | 26.4 ± 7.8* | 27.4 ± 10.6* | 29.3 ± 9.0* | < 0.001 |
| TAR1 (>180 <=250 mg/dL; >10 <=13.9 mmol/L) | 26.9 ± 5.7 | 17.4 ± 4.9* | 17.3 ± 5.3* | 18.3 ± 3.8* | < 0.001 |
| TAR2 (>250 mg/dL; >13.9 mmol/L) | 24.6 [21.5; 28.2] | 8.4 [6.3; 12.2]* | 9.6 [6.1; 12.7]* | 10.1 [6.8; 14.2]* | < 0.001 |
| ***Glycaemic targets*** |  |  |  |  |  |
| Users reaching HbA1c <=6.5% | 0/20 (0.0%) |  | 2/20 (10.0%) | 2/19 (10.5%) | .368 |
| Users reaching TIR >70% | 0/20 (0.0%) | 9/20 (45.0%)* | 10/20 (50.0%)* | 8/20 (40.0%)* | 0.002 |
| Users reaching TBR (<70 mg/dL) <4% | 14/20 (70.0%) | 13/20 (65.0%) | 11/20 (55.0%) | 16/20 (80.0%) | 0.181 |
| Users reaching TBR2 (<54 mg/dL) <1% | 13/20 (65.0%) | 14/20 (70.0%) | 15/20 (75.0%) | 16/20 (80.0%) | 0.623 |
| ***Insulin*** |  |  |  |  |  |
| Total daily dose | 45.4 ± 17.8 | 45.4 ± 17.4 | 49.9 ± 17.7 | 46.5 ± 16.1 | 0.133 |
| Basal | 20.6 ± 8.2 | 20.3 ± 9.0 | 25.4 ± 12.9 | 21.2 ± 9.4 | 0.017 |
| Bolus | 24.8 ± 11.9 | 24.9 ± 10.0 | 25.8 ± 9.5 | 25.3 ± 9.8 | 0.939 |
| Time with advanced hybrid closed loop active, % |  | 98.8 [91.5; 99.6] | 99.8 [98.5; 99.9] | 99.6 [98.1; 99.7] | 0.025 |
| ***Anthropometric measures*** |  |  |  |  |  |
| BMI Z score | 0.9 [0.1; 1.5] |  | 1.2 [0.3; 1.8]* | 1.2 [0.3; 1.7]* | 0.028 |

**Supplementary Table S3:** Longitudinal glycaemic, insulin, and anthropometric outcomes according to baseline HbA1c category. Panel A includes participants with baseline HbA1c <8% (n = 56), and Panel B includes participants with baseline HbA1c ≥8% (n = 20). Data are presented as mean ± SD for normally distributed variables and median [P25; P75] for non-normally distributed variables. Categorical outcomes are presented as n/N (%). Global p-values refer to repeated-measures ANOVA or Friedman test, as appropriate. When the global test was significant, post-hoc comparisons versus baseline were performed using Holm correction. * indicates p < 0.05 versus baseline.
