# Supplementary material for: Real‐World Effectiveness and Safety of a Tubeless Automated Insulin Delivery System With an Adaptive Control Algorithm (APGO) in Children and Adolescents With Type 1 Diabetes: A Multicentre Observational Study
Source: Diabetes Obes Metab. 2026 Jul 23;28(10):9275–83. doi: 10.1111/dom.71101 (PMC13538724; doi:10.1111/dom.71101)
Supplement: Supplementary file 4 [file DOM-28-9275-s004.docx]

| **Timepoint** | **N** | **Method** | **r** | **p** | **p adj. Holm** |
| --- | --- | --- | --- | --- | --- |
| 1 month | 76 | Pearson | 0.849 | < 0.001 | < 0.001 |
| 3 months | 76 | Pearson | 0.870 | < 0.001 | < 0.001 |
| 6 months | 76 | Pearson | 0.847 | < 0.001 | < 0.001 |

**Supplementary Table 1: Correlation between improvements in Time in Tight Range (TiTR, 70–140 mg/dL) and Time in Range (TIR, 70–180 mg/dL) from baseline to 1, 3, and 6 months.** Pearson correlation analyses were performed to assess the association between changes in TiTR and changes in TIR at each follow-up timepoint. Improvements were calculated as the difference between follow-up and baseline values. P-values were adjusted for multiple comparisons using the Holm correction method.
