# Supplementary material for: Real‐World Effectiveness and Safety of a Tubeless Automated Insulin Delivery System With an Adaptive Control Algorithm (APGO) in Children and Adolescents With Type 1 Diabetes: A Multicentre Observational Study
Source: Diabetes Obes Metab. 2026 Jul 23;28(10):9275–83. doi: 10.1111/dom.71101 (PMC13538724; doi:10.1111/dom.71101)
Supplement: Supplementary file 5 [file DOM-28-9275-s005.docx]

Supplementary Table 4A

*Glycaemic Outcomes According to Age Group: >= 12 years (N=49)*

| **Variable** | **Baseline** | **1 month** | **3 months** | **6 months** | **Global p-value** |
| --- | --- | --- | --- | --- | --- |
| Glycaemic control |  |  |  |  |  |
| HbA1c (%) | 7.5 ± 0.7 |  | 6.9 ± 0.7* | 6.9 ± 0.6* | 0.000 |
| Sensor glucose, mg/dL | 170.8 ± 29.4 | 147.6 ± 13.5* | 150.6 ± 17.2* | 154.3 ± 15.2* | 0.000 |
| Standard deviation of sensor glucose, mg/dL | 63.9 ± 17.1 | 58.1 ± 11.6 | 59.2 ± 13.9 | 60.6 ± 13.6 | 0.053 |
| Coefficient of variation, % | 38.8 ± 6.8 | 38.8 ± 6.0 | 39.1 ± 5.9 | 39.3 ± 6.6 | 0.928 |
| GMI, % | 7.4 ± 0.7 | 6.8 ± 0.3* | 6.9 ± 0.4* | 7.0 ± 0.3* | 0.000 |
| Time in ranges, % |  |  |  |  |  |
| TBR2 (<54 mg/dL; <3 mmol/L) | 0.0 [0.0; 1.0] | 0.2 [0.1; 0.8] | 0.2 [0.0; 0.8] | 0.2 [0.0; 0.4] | 0.105 |
| TBR1 (>=54 <70 mg/dL; >=3 <3.9 mmol/L) | 1.0 [0.6; 3.7] | 1.6 [1.2; 3.0] | 1.4 [0.8; 2.5] | 1.3 [0.9; 2.4] | 0.336 |
| TBR (<70 mg/dL; <3.9 mmol/L) | 1.8 [1.0; 5.2] | 1.8 [1.4; 3.9] | 1.9 [1.0; 3.5] | 1.3 [1.0; 2.8] | 0.307 |
| TITR (70-140 mg/dL; 3.9-7.8 mmol/L) | 31.1 [24.0; 41.5] | 51.1 [46.9; 55.7]* | 50.6 [45.9; 58.2]* | 47.4 [43.1; 52.1]* | 0.000 |
| TIR (70-180 mg/dL; 3.9-10.0 mmol/L) | 57.7 ± 15.5 | 73.5 ± 9.0* | 72.9 ± 10.4* | 70.8 ± 9.7* | 0.000 |
| TAR (>180 mg/dL; >10 mmol/L) | 38.6 ± 16.6 | 23.6 ± 8.6* | 24.7 ± 10.3* | 27.0 ± 9.9* | 0.000 |
| TAR1 (>180 <=250 mg/dL; >10 <=13.9 mmol/L) | 23.2 ± 8.4 | 16.7 ± 5.4* | 16.8 ± 5.6* | 17.7 ± 5.1* | 0.000 |
| TAR2 (>250 mg/dL; >13.9 mmol/L) | 16.0 [5.9; 22.4] | 6.4 [4.1; 9.9]* | 6.1 [4.2; 10.9]* | 8.2 [4.1; 11.1]* | 0.000 |
| Glycaemic targets |  |  |  |  |  |
| Users reaching HbA1c <=6.5% | 5/49 (10.2%) |  | 11/49 (22.4%) | 15/48 (31.2%)* | 0.015 |
| Users reaching TIR >70% | 11/49 (22.4%) | 34/49 (69.4%)* | 29/49 (59.2%)* | 26/49 (53.1%)* | 0.000 |
| Users reaching TBR (<70 mg/dL) <4% | 34/49 (69.4%) | 37/49 (75.5%) | 38/49 (77.6%) | 40/49 (81.6%) | 0.340 |
| Users reaching TBR2 (<54 mg/dL) <1% | 32/49 (65.3%) | 40/49 (81.6%) | 39/49 (79.6%) | 43/49 (87.8%)* | 0.013 |
| Insulin |  |  |  |  |  |
| Total daily dose | 49.0 [39.0; 56.0] | 45.3 [41.0; 53.3] | 48.1 [40.4; 54.7] | 47.5 [42.3; 53.6] | 0.169 |
| Basal | 23.0 [18.6; 29.0] | 21.5 [16.8; 27.5] | 23.5 [17.1; 27.4] | 20.7 [17.2; 27.9] | 0.130 |
| Bolus | 26.0 [17.2; 31.0] | 26.6 [18.7; 31.6] | 24.8 [20.7; 31.8] | 23.9 [20.4; 31.2] | 0.491 |
| Time with advanced hybrid closed loop active, % |  | 98.7 [93.0; 99.8] | 99.2 [96.7; 99.9] | 99.7 [96.6; 99.8] | 0.216 |
| Anthropometric measures |  |  |  |  |  |
| BMI Z score | 0.6 ± 0.8 |  | 0.6 ± 0.8 | 0.7 ± 0.8 | 0.867 |

Supplementary Table 4B

*Glycaemic Outcomes According to Age Group: < 12 years (N=27)*

| **Variable** | **Baseline** | **1 month** | **3 months** | **6 months** | **Global p-value** |
| --- | --- | --- | --- | --- | --- |
| Glycaemic control |  |  |  |  |  |
| HbA1c (%) | 7.1 [6.8; 7.9] |  | 7.0 [6.6; 7.3]* | 6.8 [6.4; 7.2]* | 0.004 |
| Sensor glucose, mg/dL | 167.0 ± 31.5 | 144.1 ± 15.2* | 147.3 ± 14.8* | 147.0 ± 15.0* | 0.000 |
| Standard deviation of sensor glucose, mg/dL | 63.2 ± 16.5 | 58.8 ± 15.8 | 58.9 ± 13.8 | 57.1 ± 13.8 | 0.076 |
| Coefficient of variation, % | 37.5 ± 4.8 | 39.9 ± 7.9 | 39.6 ± 6.8 | 39.2 ± 6.6 | 0.067 |
| GMI, % | 7.3 ± 0.8 | 6.7 ± 0.4* | 6.8 ± 0.4* | 6.8 ± 0.4* | 0.000 |
| Time in ranges, % |  |  |  |  |  |
| TBR2 (<54 mg/dL; <3 mmol/L) | 0.0 [0.0; 0.8] | 0.4 [0.0; 0.6] | 0.3 [0.1; 0.8] | 0.2 [0.0; 0.6] | 0.240 |
| TBR1 (>=54 <70 mg/dL; >=3 <3.9 mmol/L) | 1.9 [1.0; 3.0] | 2.7 [1.4; 4.2] | 2.0 [0.9; 3.5] | 2.2 [0.9; 2.6] | 0.285 |
| TBR (<70 mg/dL; <3.9 mmol/L) | 2.1 [1.0; 4.0] | 3.2 [1.4; 5.0] | 2.5 [0.9; 4.3] | 2.4 [1.1; 3.2] | 0.447 |
| TITR (70-140 mg/dL; 3.9-7.8 mmol/L) | 38.6 ± 14.2 | 55.4 ± 10.6* | 52.9 ± 9.9* | 52.4 ± 9.0* | 0.000 |
| TIR (70-180 mg/dL; 3.9-10.0 mmol/L) | 63.0 [49.0; 74.8] | 74.9 [68.3; 80.2]* | 71.5 [67.2; 80.6]* | 75.3 [67.6; 78.6]* | 0.000 |
| TAR (>180 mg/dL; >10 mmol/L) | 35.0 [22.1; 48.5] | 20.6 [17.3; 28.4]* | 24.3 [15.4; 30.1]* | 23.5 [17.4; 30.8]* | 0.000 |
| TAR1 (>180 <=250 mg/dL; >10 <=13.9 mmol/L) | 22.2 ± 6.3 | 14.6 ± 5.2* | 15.9 ± 5.0* | 16.7 ± 4.6* | 0.000 |
| TAR2 (>250 mg/dL; >13.9 mmol/L) | 9.0 [3.8; 22.5] | 5.9 [3.2; 10.2]* | 7.5 [2.8; 11.2]* | 6.1 [2.9; 10.5]* | 0.001 |
| Glycaemic targets |  |  |  |  |  |
| Users reaching HbA1c <=6.5% | 5/27 (18.5%) |  | 6/27 (22.2%) | 12/27 (44.4%) | 0.037 |
| Users reaching TIR >70% | 10/27 (37.0%) | 17/27 (63.0%) | 18/27 (66.7%) | 17/27 (63.0%) | 0.037 |
| Users reaching TBR (<70 mg/dL) <4% | 20/27 (74.1%) | 16/27 (59.3%) | 19/27 (70.4%) | 22/27 (81.5%) | 0.236 |
| Users reaching TBR2 (<54 mg/dL) <1% | 20/27 (74.1%) | 22/27 (81.5%) | 24/27 (88.9%) | 24/27 (88.9%) | 0.348 |
| Insulin |  |  |  |  |  |
| Total daily dose | 26.0 [20.8; 32.5] | 26.6 [20.9; 30.9] | 26.1 [20.9; 31.8] | 27.0 [19.3; 34.1] | 0.308 |
| Basal | 13.0 [10.8; 16.7] | 11.2 [7.8; 13.3]* | 11.3 [9.1; 15.3] | 12.0 [8.6; 16.1] | 0.001 |
| Bolus | 13.0 [9.0; 15.4] | 14.1 [11.9; 18.1]* | 13.8 [12.2; 17.9]* | 15.1 [11.2; 19.0]* | 0.007 |
| Time with advanced hybrid closed loop active, % |  | 99.6 [94.2; 99.8] | 99.7 [98.8; 99.9] | 99.7 [98.3; 100.0] | 0.399 |
| Anthropometric measures |  |  |  |  |  |
| BMI Z score | 0.8 [0.2; 1.4] |  | 0.8 [0.1; 1.8] | 0.9 [0.0; 1.6] | 0.598 |

**Supplementary Table S4:** Longitudinal glycaemic, insulin, and anthropometric outcomes according to age group. Panel A includes participants aged ≥12 years (n = 49), and Panel B includes participants aged <12 years (n = 27). Data are presented as mean ± SD for normally distributed variables and median [P25; P75] for non-normally distributed variables. Categorical outcomes are presented as n/N (%). Global p-values refer to repeated-measures ANOVA or Friedman test, as appropriate. When the global test was significant, post-hoc comparisons versus baseline were performed using Holm correction. * indicates p < 0.05 versus baseline.
