# Supplementary material for: Real‐World Effectiveness and Safety of a Tubeless Automated Insulin Delivery System With an Adaptive Control Algorithm (APGO) in Children and Adolescents With Type 1 Diabetes: A Multicentre Observational Study
Source: Diabetes Obes Metab. 2026 Jul 23;28(10):9275–83. doi: 10.1111/dom.71101 (PMC13538724; doi:10.1111/dom.71101)
Supplement: Supplementary file 6 [file DOM-28-9275-s006.docx]

| **Variable** | **Timepoint** | **< 12 years** | **>= 12 years** | **p** | **p adj.** |
| --- | --- | --- | --- | --- | --- |
| ΔHbA1c | 3 months | -0.36 ± 0.94 (n=27) | -0.57 ± 0.80 (n=49) | 0.329 | 1.000 |
| ΔHbA1c | 6 months | -0.41 ± 0.85 (n=27) | -0.55 ± 0.67 (n=49) | 0.453 | 1.000 |
| ΔTIR | 1 month | 14.05 ± 9.53 (n=27) | 15.84 ± 14.39 (n=49) | 0.518 | 1.000 |
| ΔTIR | 3 months | 12.62 ± 12.97 (n=27) | 15.19 ± 14.74 (n=49) | 0.435 | 1.000 |
| ΔTIR | 6 months | 12.16 ± 12.52 (n=27) | 13.17 ± 12.95 (n=49) | 0.742 | 1.000 |
| ΔTiTR | 1 month | 16.85 ± 8.96 (n=27) | 18.15 ± 11.92 (n=49) | 0.594 | 1.000 |
| ΔTiTR | 3 months | 14.28 ± 11.01 (n=27) | 17.25 ± 12.62 (n=49) | 0.290 | 1.000 |
| ΔTiTR | 6 months | 13.78 ± 12.91 (n=27) | 14.27 ± 11.43 (n=49) | 0.870 | 1.000 |

**Supplementary Table S5:** Between-group comparisons of changes in HbA1c, Time in Range (TIR, 70–180 mg/dL), and Time in Tight Range (TiTR, 70–140 mg/dL) according to age group (<12 years vs ≥12 years). Delta (Δ) values were calculated as follow-up minus baseline. Values are presented as mean ± SD for normally distributed variables and median [P25; P75] for non-normally distributed variables. p-values refer to between-group comparisons, and adjusted p-values were calculated using the Holm correction method. Sample size for each comparison is reported in parentheses. No significant differences in ΔHbA1c, ΔTIR, or ΔTiTR were observed between age groups at any follow-up timepoint.
